# Effectiveness of Consumers Washing with Sanitizers to Reduce Human Norovirus on Mixed Salad

**DOI:** 10.3390/foods8120637

**Published:** 2019-12-03

**Authors:** Eduard Anfruns-Estrada, Marilisa Bottaro, Rosa M. Pintó, Susana Guix, Albert Bosch

**Affiliations:** 1 Enteric Virus Laboratory, Department of Genetics, Microbiology and Statistics, University of Barcelona, 08028 Barcelona, Spain; eanfruns@ub.edu (E.A.-E.); marilisa.bottaro@uniba.it (M.B.); rpinto@ub.edu (R.M.P.); abosch@ub.edu (A.B.); 2 Nutrition and Food Safety Research Institute (INSA·UB), University of Barcelona, Santa Coloma de, 08921 Gramenet, Spain; 3 Department of Veterinary Medicine, University of Bari Aldo Moro, 70010 Bari, Italy

**Keywords:** human norovirus, leafy greens, disinfection, chlorine, peracetic acid, PMAxx-viability RTqPCR

## Abstract

Human norovirus (HuNoV) is a foremost cause of domestically acquired foodborne acute gastroenteritis and outbreaks. Despite industrial efforts to control HuNoV contamination of foods, its prevalence in foodstuffs at retail is significant. HuNoV infections are often associated with the consumption of contaminated produce, including ready-to-eat (RTE) salads. Decontamination of produce by washing with disinfectants is a consumer habit which could significantly contribute to mitigate the risk of infection. The aim of our study was to measure the effectiveness of chemical sanitizers in inactivating genogroup I and II HuNoV strains on mixed salads using a propidium monoazide (PMAxx)-viability RTqPCR assay. Addition of sodium hypochlorite, peracetic acid, or chlorine dioxide significantly enhanced viral removal as compared with water alone. Peracetic acid provided the highest effectiveness, with log_10_ reductions on virus levels of 3.66 ± 0.40 and 3.33 ± 0.19 for genogroup I and II, respectively. Chlorine dioxide showed lower disinfection efficiency. Our results provide information useful to the food industry and final consumers for improving the microbiological safety of fresh products in relation to foodborne viruses.

## 1. Introduction

Human noroviruses (HuNoVs), members of the family *Caliciviridae*, are recognized as a leading cause of outbreaks of acute viral gastroenteritis and foodborne illness worldwide, affecting all age groups. Globally, HuNoVs account for 18% of all cases of acute gastroenteritis [1] and 18% of all foodborne illnesses [2]. The general population is broadly vulnerable to HuNoV disease across all age groups, but the majority of morbidity and mortality occurs at the extremes of age. The fecal–oral route is the main mode of transmission, although several other modalities have been described. These modalities include transmission via aerosolized viral particles in vomitus, environmental contamination, and through food and water, chiefly shellfish, soft fruit, and vegetables [3,4]. Fresh produce such as leafy greens and fruits contaminated with HuNoV have become increasingly recognized as potential vehicles of HuNoV transmission; being increasingly reported as a causative agent in a high proportion of outbreaks in many parts of the world in the last years [5,6,7]. In addition, HuNoV was reported in 29% of all alerts reported in the Rapid Alert System for Food and Feed (RASFF; https://ec.europa.eu/food/safety/rasff_en) system in 2017 on fruits and vegetables. 

HuNoVs may contaminate fruits and vegetables at any point of the production chain, including cultivation before harvest or post-harvest. Contamination by an infected food handler and exposure to fecally contaminated water during irrigation are thought to be the most frequent modes of HuNoV contamination of foods moving through the farm-to-fork continuum [8,9]. They are able to survive outside the host and remain relatively stable under food processing and storage conditions (reviewed by [10]). Of note, in 2010, vegetables contaminated with HuNoV, especially lettuce, accounted for 50% of the outbreaks attributed to vegetables, fruits, and products thereof which were reported in Europe [11], and the importance of contaminated vegetables as the food vehicle implicated in a significant number of 2009–2012 outbreaks in the United States has also been reported [12]. Although Baert et al. (2011) [13] reported notably high HuNoV prevalence in leafy greens during 2009–2010 in Belgium and France, most recent screenings during 2011–2017 from Italy and the UK report lower prevalence, ranging from 0–5% (reviewed by [14,15]), but the number of studies may be insufficient to determine whether a change over the years is occurring. Additionally, leafy green contamination may also depend on the epidemiology of HuNoV in the population during that period.

Preventative processing strategies are promising means to reduce occurrence of HuNoV contamination at retail in such products, or at least reduce viral loads, and washing of harvested produce is a common practice in the food industry. Decontamination of produce using different disinfectants has been evaluated [16,17,18,19], but due to the high variability of food matrices and the recent development of novel capsid integrity assays which provide a better correlation with infectivity [20,21], new investigations and intervention measures in the food chain of vegetables still need to be evaluated for their efficacy in viral inactivation.

The purpose of our study was to evaluate the effectiveness of different chemical agents with a potential to be used domestically, in inactivating HuNoVs genogroup I (GI) and genogroup II (GII) strains on vegetables purchased from Spanish supermarkets, using a viability RTqPCR assay to avoid detection of noninfectious viral particles and obtain a better assessment of infectivity reduction.

## 2. Materials and Methods

### 2.1. Human Noroviruses

Stool specimens positive for HuNoV GI and GII were obtained from patients with gastroenteritis from outbreaks declared to the Public Health Agency of Catalonia (kindly provided by R. Bartolomé from the Hospital Universitari Vall d’Hebron, Spain) and were used as HuNoV reference material. HuNoV genotypes were identified as GI.3 and GII.2, as previously described [22]. HuNoV stool samples were suspended (10%, wt/vol) in phosphate-buffered saline (PBS) buffer and pelleted at 10,000× *g* for 5 min. Fecal suspension was not filtered in order to better mimic what would occur under natural conditions. The supernatant was stored at −80 °C in aliquots. These supernatants were retained for RNA extraction and RTqPCR quantification, following the protocol described at the ISO 15216-1:2017 [23]. Briefly, RNA was extracted using the NucliSens^®^ miniMAG magnetic system (BioMérieux, France) following the manufacturer’s instructions, and viral genomes were quantitated by a duplex RTqPCR reaction using the previously validated primers and probes [23] and using the RNA UltraSense One-Step quantitative RT-PCR system (Invitrogen, Calsbad, CA, USA) and the Strategene Mx3000P system. Forward primer, reverse primer, and probe concentrations were 500, 900, and 250 nM, respectively. Cycling parameters for duplex RTqPCR assays were 1 h at 55 °C, followed by 5 min at 95 °C, and 45 cycles of 15 s at 95 °C, 1 min at 60 °C, and 1 min at 65 °C. The standard curves were obtained using double-stranded synthetic DNA containing the corresponding target GI and GII sequences.

### 2.2. Spiking of NoV GI and GII onto Fresh Produce

Ready-to-eat (RTE) mixed salad samples (containing 82% iceberg lettuce, 10% red cabbage, and 8% sliced carrots) were obtained from a local supermarket. Then, 25 g of sample was spiked with 100 μL of the appropriate dilution of each viral stock solution to obtain 8 × 10^6^ genome copies for GI and 1.2 × 10^7^ genome copies for GII. Each inoculum was spotted by using a micropipette with about 50 to 100 drops on the surface of each sample. The inoculum was allowed to adsorb for 1 h under a laminar flow hood at room temperature (RT) to ensure the drying and adhesion of the viral particles. Non-inoculated mixed salad samples were used as negative controls to exclude the occurrence of natural contamination of the samples.

### 2.3. Chemical Disinfection Treatment

Sanitizers concentrations tested were selected based on label indications and FDA (Food and Drug Administration) recommendations. Household bleach was used as a source of sodium hypochlorite, following the manufacturer’s instructions for produce disinfection. Peracetic acid (PAA) was obtained from Merck Millipore, and chlorine dioxide (DK-DOX^®^ AGRAR) was purchased from APURA (Gargnano, Italy). Sodium hypochlorite was tested at 100 ppm, peracetic acid at 80 ppm, and chlorine dioxide at 20 ppm. Each disinfectant solution was freshly prepared for each experiment at the desired concentrations in tap water (pH 7.3; conductivity 1000 µS/cm), to better mimic what would occur in domestic kitchens and households. Spiked samples were gently immersed in each disinfectant solution for 10 min without agitation at RT, and samples washed in parallel with tap water only were also included. After treatment, the food surface was rinsed with 500 mL of tap water before the virus recovery steps. Inoculated and unwashed samples were used as positive controls.

### 2.4. Sample Processing for Virus Extraction and Quantification

Virus extraction was performed following the ISO 15216-1:2017 [23]. Limit of detection of the analysis of HuNoV on lettuce using the ISO 15216-1:2017 was of 0.46 and 0.88 copies/g for GI and GII, respectively [24]. Briefly, 25 g of sample was added to 40 mL of Tris/glycine/beef extract (TGBE) buffer pH 9.5 (100 mM Tris–HCl, 50 mM glycine, 1% beef extract) and 10 μL of Mengovirus as a process control virus material. The eluate was concentrated with 5× polyethylene glycol (PEG)/NaCl solution (50% (w/v) PEG 8000, 1.5 M NaCl). Viruses were concentrated by centrifuging the solution at 10,000× *g* for 30 min at 4 °C. The pellet was re-suspended in 500 μL of PBS. Viral genome quantification was performed by propidium monoazide (PMAxx)-viability RTqPCR assay, as previously described [20,25], with minor modifications. Briefly, sample extracts were incubated with 50 μM PMAxx (Biotinum) and 0.5% Triton X-100 in the dark at RT for 10 min at 150 rpm. Samples were then exposed to light for 15 min using a photo activation system (Led-Active Blue, Geniul) and subsequently extracted using the NucliSens^®^ miniMAG magnetic kit (BioMérieux) according to the manufacturer’s instructions. HuNoV GI and GII genomes were quantitated by a duplex RTqPCR reaction as described above. For each sample, extraction efficiency was monitored by performing a monoplex RTqPCR assay using Mengovirus specific primers, and RT-PCR inhibition was assessed using an external control RNA, as described [23].

### 2.5. Data Analysis

Each condition was assayed in three–four independent biological experiments performed on separate days and performed by two different workers. Viruses from each sample were extracted and titrated by RTqPCR in duplicate wells. Effect of the sanitizer wash on virus reduction was determined by calculating the log units (N_t_/N_0_), where N_0_ is the titer of virus recovered on unwashed control samples and N_t_ is the titer of virus recovered on washed sample. Differences between sanitizer types or genogroups were determined by one-way analysis of variance (ANOVA) (*p* < 0.05), using SPSS Statistics 22 (IBM, Armonk, NY, USA)

## 3. Results

**Figure 1:**
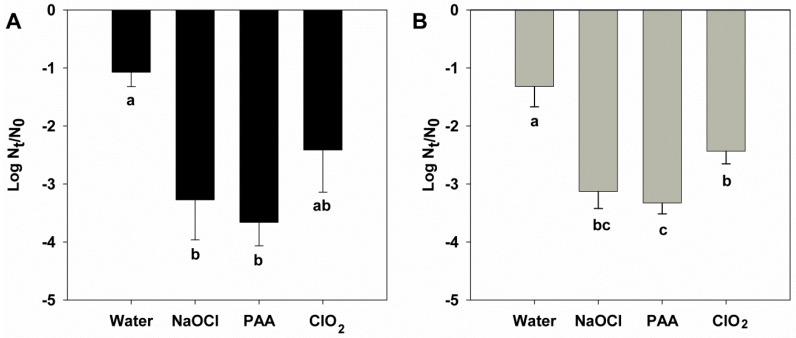
Reduction of human norovirus (HuNoV) genogroup I (GI) (**A**) and genogroup II (GII) (**B**) genomes on salad, as measured by PMA-RTqPCR, after 10 min wash with water alone, sodium water containing sodium hypochlorite (NaOCl) at 100 ppm, peracetic acid (PAA) at 80 ppm, and chlorine dioxide (ClO_2_) at 20 ppm. Each data bar represents an average of three–four independent experiments, and error bar shows the standard deviation. Different letters indicate a significant (*p* < 0.05) effect between treatments for each genogroup. N_0_: original virus titer; N_t_: virus titer after treatment.

Results obtained of HuNoV removal during washing procedures are shown in Figure 1. A 10 min washing step with tap water alone reduced viral titers for both HuNoV GI and GII by 1.08 ± 0.25 and 1.32 ± 0.35 log_10_, respectively. The three tested disinfectants significantly enhanced the removal of both genogroups, as compared with washing with water alone. Sodium hypochlorite and PAA showed the highest removal efficiencies, which were similar for both genogroups, adding approximately 2 log_10_ inactivation in all cases as compared with water. Removal/disinfection efficiencies observed for GI and GII were not statistically different. PAA provided the highest effectiveness, with log_10_ reductions on virus levels of 3.66 ± 0.40 and 3.33 ± 0.19 for GI and GII, respectively. Since all measurements were performed including a PMAxx treatment prior to RTqPCR, final titers correspond to viruses with undamaged capsids. 

The following parameters were measured to control the performance of the method. Average percentage recovery of Mengovirus process control virus was 40.6% ± 44.9%. Average percentage recoveries of inoculated HuNoV GI and GII were 15.3% ± 11.6% and 22.7% ± 16.8%, respectively. Average percentages of RTqPCR efficiencies as measured by external RNA controls for GI and GII were 60.7% ± 16.6% and 65.1% ± 26.1%, respectively. No changes in the aspect of mixed salads were noticed with any of the treatments conducted.

None of the 30 non-inoculated RTE samples tested positive for HuNoV GI and GII, suggesting a low prevalence of naturally contaminated samples in RTE salads at retail.

## 4. Discussion

The aim of this study was to assess the efficacy of chemical disinfectants (sodium hypochlorite, peracetic acid, and chlorine dioxide) at decontaminating vegetables spiked with HuNoV GI and GII, using a viability RTqPCR assay to obtain a better assessment of infectivity reduction. The disinfectants used in this study are on the list approved by the FDA for the washing of fruits and vegetables, and their effectiveness and limitations have been extensively studied [26,27]. They were also chosen for our study based on their potential ease of their domestic use. Sodium hypochlorite is popularly used as a produce wash water sanitizer due to its documented efficacy and low cost, but it produces unhealthy by-products and its efficiency in disinfection is largely reduced by the presence of organic matter. Chlorine and its derivatives have, however, been in the spotlight due to environmental concerns. Certain organic acids such as PAA are frequently proposed as “natural” approaches to prevent microbial contamination. In the fresh-cut industry, PAA is often used as a commercial sanitizer in combination with other organic acids and hydrogen peroxide, producing acceptable microbial mortality levels. Finally, chlorine dioxide has received more attention as a decontaminant for vegetables and fruits because it is less affected by pH, it does not form organohalogen by-products, and its oxidative power is stronger than that of sodium hypochlorite [27].

Sensitivity of noroviruses to chlorine and other sanitizers has been proven on different food matrices using the murine norovirus surrogate measured by infectivity assays as well as human viruses measured by viral genome reduction [10,14], but very few studies have used viability RTqPCR assays to better estimate the achieved reduction in HuNoV infectivity [28].

Suitability of PMAxx-RTqPCR for discrimination between potentially infectious enteric viruses and viruses with structural damages caused by chemical disinfectants has been confirmed by others and us [25,28,29,30], but these assays have not been extensively used yet on viral inactivation studies. Although the human intestinal enteroid model [31] would be the best tool to determine the efficacy of inactivation procedures [32], the use of this system on a routine basis to provide quantitative estimates of log_10_ reduction levels is still unaffordable for most laboratories.

Our results show that while reductions observed by PMAxx-RTqPCR assay after washing with water alone were similar to previously reported data (which range between 0.1 and 1.8 log_10_) [33,34], total reductions observed with sodium hypochlorite (100 ppm) and PAA (80 ppm) consistently reached over 3 log_10_ reduction, which is higher than what has been published by other studies using similar disinfection treatments on other enteric viral targets when measured by conventional RTqPCR assays [16,17,35]. We believe that the higher log reductions observed in our study may be primarily explained by the longer exposure time used (10 min), but also the use of PMAxx-RTqPCR assay to measure only recovered viruses with intact capsids. Although our data cannot confirm whether the disinfection treatments performed rendered some viruses noninfectious due to structural damages, we show that sodium hypochlorite (100 ppm) or PAA (80 ppm) may ensure a complete HuNoV elimination from mixed salad samples if the virus is present in numbers lower than 3 log_10_. Once sufficient data on viral load occurring on contaminated produce products are gathered, it will be possible to assess whether this level of viral removal can be regarded as a safe virucidal objective in terms of risk mitigation. Of interest, PAA, which has a growing popularity from a safety and environmental perspective, showed an efficacy similar to sodium hypochlorite, confirming its potential as an alternative to chlorine [27]. Of note, PAA has also been proven to be more effective than sodium hypochlorite and chloride dioxide on lettuce to remove murine norovirus [17]. Finally, our results also showed similar behaviors for both HuNoV genogroups. Differences with previous observations showing a higher resistance of GII to free chlorine [18] or chlorine dioxide [28] may be partially explained by the use of different viral genotypes or different types of matrices. In addition, the study performed by Dunkin et al. [18] did not use the PMAxx-RTqPCR assay, which avoids detection of viruses with compromised capsids.

As a conclusion, our data confirm the suitability of sodium hypochlorite and especially PAA as disinfectants to be applied in the fresh-cut industry, in combination with an optimal management of hygiene and control of other critical points of possible contamination defined by Hazard Analysis and Critical Control Points (HACCP) plans. Since both products may also be potentially used in domestic households, as well as in catering services and in restoration, their implementation as produce chemical disinfectants should be reinforced in these settings, in order to mitigate the risk of HuNoV infections when contamination at retail is not completely prevented.
